# The interplay of contextual elements in implementation: an ethnographic case study

**DOI:** 10.1186/s12913-015-0713-7

**Published:** 2015-02-14

**Authors:** Megan B McCullough, Ann F Chou, Jeffrey L Solomon, Beth Ann Petrakis, Bo Kim, Angela M Park, Ashley J Benedict, Alison B Hamilton, Adam J Rose

**Affiliations:** VA HSR&D Center for Healthcare Organization and Implementation Research, ENRM Veterans Hospital, Bedford, MA USA; The University of Oklahoma Health Sciences Center, Oklahoma City, OK USA; VA New England Healthcare System, Bedford, MA USA; VA Sunshine Healthcare Network, St. Petersburg, FL USA; VA HSR&D Center for the Study of Healthcare Innovation, Implementation and Policy, VA Greater Los Angeles, Los Angeles, CA USA; University of California, LA, Los Angeles, CA USA; Boston University School of Medicine, Boston, MA USA

**Keywords:** Organizational context, Contextual elements, Quality improvement, Interplay, PARIHS, Implementation

## Abstract

**Background:**

Contextual elements have significant impact on uptake of health care innovations. While existing conceptual frameworks in implementation science suggest contextual elements interact with each other, little research has described how this might look in practice. To bridge this gap, this study identifies the interconnected patterns among contextual elements that influence uptake of an anticoagulation clinic improvement initiative.

**Methods:**

We completed 51 semi-structured interviews and ethnographic observations across five case study sites involved in an evidence-based practice (EBP) quality improvement initiative. We analyzed data in NVivo 10 using an *a priori* approach based on the Promoting Action on Research Implementation in Health Services (PARIHS) model and an emergent thematic analysis.

**Results:**

Key contextual elements, such as leadership, teamwork, and communication, interacted with each other in contributing to site-level uptake of the EBP, often yielding results that could not be predicted by looking at just one of these elements alone. Sites with context conducive to change in these areas predictably had high uptake, while sites with uniformly weak contextual elements had low uptake. Most sites presented a mixed picture, with contextual elements being strongly supportive of change in some areas and weak or moderate in others. In some cases, we found that sites with strong context in at least one area only needed to have adequate context in other areas to yield high uptake. At other sites, weak context in just one area had the potential to contribute to low uptake, despite countervailing strengths. Even a site with positive views of EBPs could not succeed when context was weak.

**Conclusion:**

Interrelationships among different contextual elements can act as barriers to uptake at some sites and as facilitators at others. Accounting for interconnections among elements enables PARIHS to more fully describe the determinants of successful implementation as they operate in real-world settings.

## Background

The uptake of evidence-based practices (EBPs) and quality improvement (QI) interventions can be notoriously challenging. The effort to implement EBPs entails attention not only to designing an appropriate intervention, but also to the dynamic organizational context in which the intervention is to be implemented. Established implementation science (IS) work on barriers and facilitators to uptake has proven useful in real-world implementation efforts, especially in regard to identifying individual contextual elements and noting ways these singular contextual components influence the implementation of health care innovations [1-6].

Implementation researchers anticipate that when a majority of contextual elements are conducive to change, implementation is usually successful. In contrast, when a majority of contextual elements are not conducive to change, implementation is not successful. However, in many cases where EBPs or QI projects are implemented, there is a more mixed, intermediate picture that can produce interesting, yet unpredictable, patterns of uptake. Our study identifies and describes a range of such situations at five sites with a “mixed” picture of contextual elements. Therefore in this study we discuss more broadly how interactions of contextual elements impact QI projects. Since little is known about these interactions [7], the present study contributes to the IS and QI literature by describing how interplay among contextual elements can influence the uptake of change. In this paper, we use the term “uptake” to indicate the initiation, incorporation and use of an EBP or health care innovation.

### Conceptual framework and contextual factor definitions

We used the Promoting Action on Research Implementation in Health Services (PARIHS) model prospectively to implement a large anticoagulation improvement project. PARIHS states that successful implementation (SI) is a function (f) of the nature and type of evidence (E), the qualities of the context (C) where the evidence is being employed and the way the process is facilitated (F) or as Kitson et al. represent it, SI = f (E,C,F) [8]. Evidence includes codified and non-codified sourced of knowledge about the EBP, such as research evidence, clinical experience and local information [8]. Context includes aspects of the culture, leadership and evaluation at the site level. Facilitation refers to how an EBP is introduced and supported in clinical practice.

Context is dynamic, multivalent, and highly variable in organizational life. Key work on PARIHS recognizes the dynamic relationship among elements and sub-elements and therefore PARIHS contains within it an understanding that the same intervention will likely vary widely in different settings [9,10]. However, Helfrich et al. note that PARIHS would be strengthened if one could describe how contextual element dynamics emerge and whether or not there are some generalizable interactional patterns [9]. In the present empirical study, we identified patterns of contextual interplay and analyzed them to shed light on how such interactions operate in practice and therefore influence intervention uptake.

PARIHS elements are often described on a continuum which ranks their “strength” in regard to support for the intervention from low to high [9]. This assignation of value requires more explanation than is typically provided in implementation studies. For example, contextual elements are often ranked as to whether or not they support the proposed intervention. Yet approaching the “measurement” of contextual strength in this way may not fully capture the fact that in practice, some elements may have a mixed impact, promoting SI in some ways, but preventing it in others. Furthermore this approach to ranking contextual element strength implies that there is a linear, static relationship among contextual elements which is at odds with the dynamism implicit in the PARIHS model [9,10]. In this study we address both the strength of contextual elements as well as their effects. Therefore, the present study thus has the potential to extend and deepen PARIHS as an implementation framework [1,2,9,11,12].

### Contextual factor definitions

Recognizing that contextual elements are multidimensional, we characterize each element here. *Evidence* is the stakeholder’s perceptions of the quality and validity of the evidence supporting the intervention, in this case the ACC treatment algorithm, the dashboard that measures TTR and work processes that increase standardization. In this paper, a leader refers to those in positions of management such as Pharmacy Service Line Chiefs and middle managers (e.g., Associate Chiefs of Pharmacy). Leadership is an element of Context in PARIHS. In this study, *leadership* has three dimensions, where it: (1) describes the activities (such as supporting effective teamwork) and attitudes taken by leaders (those in management positions) to respond to the ACCII as documented by the research and implementation teams, (2) includes the perceptions staff have of their leadership’s actions and behaviors in response to the ACCII, and (3) refers to the way leaders represent themselves and their responses to the ACCII in interviews. Teamwork is a contextual factor that is embedded within the PARIHS constructs of culture and leadership. In this study, *teamwork* has two dimensions: (1) the ethnographically observed behaviors and actions that indicate whether a cooperative, coordinated effort by the ACC team is present and the extent of its presence in regard to the ACCII, and (2) the perception ACC staff have of the working relationships among their fellow ACC staff and the general staff attitude toward the ACCII. Communication is a contextual factor that is embedded in the PARIHS contextual constructs of Receptive Context, Leadership and Evaluation and Feedback. For this project, *communication* is understood as the imparting of information and meaning through speech, technology, body language, and signs. Communication also has several dimensions, referring to (1) communication from leadership to ACC team about the ACCII, and (2) the quality and strength of intra-ACC team communication about ACCII and about ACC care.

However, characterizing contextual elements only in terms of their relative strength (conducive or not conducive to change) relative to SI, while useful, is not sufficient. In fact, contextual elements can have mixed effects, or their effects can vary over time. In this study we do rank the strength of contextual elements (as defined in Table 1) in regard to how supportive and accepting they are of the ACCII intervention (Table 2), but we also use a separate table to measure and account for the positive, neutral or negative impact the element has on the ACCII or the capacity of the element to impact improvement (Table 3). In this study, we understand *interaction* to be a relationship in which two or more contextual elements may affect one another, as opposed to referring to an interaction among people. These effects are not unidirectional but rather multi-directional. Interplay can be understood as reciprocal action, interaction and the mutual influencing that occurs among contextual elements. The present article is an attempt to capture and understand these additional dimensions of how contextual features may work to promote SI.

**Table 1:** **Characteristics and ranking for strong, moderate and weak contextual elements in relation to ACCII**

	**Strong contextual elements**	**Moderate contextual elements**	**Weak contextual elements**
Evidence	*The ACC dosing algorithm is valid and the evidence for use is compelling	*The dosing algorithm used inconsistently	*Little use of the algorithm
	*The dashboard is used regularly to address loss to follow-up and patients who need more attention	*Dashboard is used inconsistently and/or only for loss to follow-up	*Dashboard rarely used
Teamwork	*Good working relations	*Mediocre working relations—not clearly working toward a common goal	*Divided teams or non-functional teams
	*Ability to problem solve together	*Divided team	*Poor working relationships
	*Team system in place to support each other	*Problem solving uneven	*Weak systems that provide little support
	*Working together to a common goal	*Mediocre system of support	*Little common effort toward working toward a common goal
Communication	*Established effective communication pathways both formal and informal	*Moderately established and used communication pathways	*Dysfunctional communication pathways both formal or informal
	*Consistent pathways for new information to spread	*Inconsistent pathways for new information to spread	*Dysfunctional pathways for new information to spread
Leadership	*Supports and leads effective teamwork	*New to leadership or new to the VA	*Not supportive of effective teamwork
	*Inclusive decision making	*Uneven use of empowerment in learning and managing	*Disempowering environment for staff
	*empowering learning and managing	*Less inclusive decision making	*Lack of role clarity roles
	*Role clarity	*Less role clarity	*Low of interaction with staff
	*Transformational leadership

**Table 2:** **Strength of contextual elements in regard to support of the ACCII and rates of uptake**

**Site**	**Evidence**	**Teamwork**	**Communication**	**Leadership**	**Uptake**
A	Strong	Strong	Strong	Moderate	High
B	Moderate	Moderate	Moderate	Strong	High
C	Moderate	Strong	Strong	Moderate	Medium
D	Weak	Weak	Moderate	Moderate	Low
E	Strong	Weak	Weak	Weak	Low

**Table 3:** **Impact/effect of contextual elements on uptake**

	**Site A**	**Site B**	**Site C**	**Site D**	**Site E**
Evidence	+	0	0	-	0
Teamwork	+	0	+	-	-
Communication	+	0	+	-	-
Leadership	0	+	-	0	-
Cumulative impact on uptake	+	+	0	-	-

Key:

Positive impact/effect on ACCII uptake = +.

Neutral impact/effect on ACCII uptake = 0.

Negative impact/effect on ACCII uptake = -.

### The intervention

In 2012, the Anticoagulation Clinic Improvement Initiative (ACCII) was introduced in a Veterans Integrated Service Network (VISN), based in the northeastern United States. The ACCII’s focus is all pharmacy-run anticoagulation clinics and all anticoagulation clinical staff in the VISN, encompassing eight medical centers and their affiliated outpatient clinics.

The most commonly used anticoagulant in the United States is warfarin, also known by the brand name Coumadin [13]. While warfarin is effective, its management is complex. The safety and effectiveness of warfarin is improved when patients spend a greater proportion of time in the therapeutic range. Anticoagulation control with warfarin is a measure of the proportion of time patients spend protected from blood clots while not being exposed to excessive risk of bleeding [14]. Anticoagulation control can be measured using percent time in therapeutic range (TTR), which has been used as a measure of control of therapy at the patient level, and of quality of care at the site level [14,15].

To improve anticoagulation care, clinical pharmacists in the VISN have been asked to adopt a dosing algorithm, an EBP, which has been shown to improve anticoagulation control and reduce rates of patient complications [16,17]. Second, ACC staff has been tasked with adopting a number of other processes of care which have also been shown to improve TTR, including prompter follow-up after out-of-range values, use of guideline-concordant target ranges, and efforts to reduce loss to follow-up [18-20]. This group of process of care measures is being monitored in real-time using a dashboard, which allows pharmacists not only to track their progress on these performance measures, but also in many cases to locate patients in need of intervention. The dashboard also measures patient-level and site-level TTR.

The ACCII is delivered by two overlapping teams. The core facilitation team at the VISN level consists of a clinical pharmacist with training in facilitation, anticoagulation care and QI, an industrial engineer, and an executive level leader, who is a QI specialist. This team is delivering and facilitating the intervention to each medical center and all satellite ACCs. The research team is studying the entire process of implementation, aggregating data on the implementation, and feeding back information to the core team to assist in tailoring and facilitating uptake. After each site visit, the research team quickly processed interim data to identify any barriers to SI which the research team then fed back to the core implementation team for tailoring their facilitation efforts. The extended team involves both the core facilitation team and research team.

## Methods

The present study focused on five ACC sites. Our cross-site comparison combined a case-oriented approach and a variable-oriented approach [21] to better capture interactional data. Using a case-oriented approach, we analyzed the data for each site for within site variation and the emergence of interactional themes. Drawing on PARIHS and inductive analysis, we identified leadership, teamwork and communication as key contextual elements at all sites in our study. We analyzed the interactions among these three contextual elements, and selected five sites for the present study whose patterns of interaction were informative or noteworthy. Working with data from these five sites, we engaged in a cross-case comparison of the qualitative data to develop case studies on the interplay of contextual elements in implementation.

### Sample and data collection

Data was collected in the latter half of 2012 and early part of 2013 (October 2012 – January 2013) and analyzed in 2013 (February – May 2013). All frontline anticoagulation pharmacy staff in the VISN was eligible to participate in the study of the ACCII by the research team. We specifically requested participation from clinical pharmacists, pharmacy technicians and medical clerks who worked a majority and/or a regular amount of time in the anticoagulation clinic. The purposive sample included pharmacy administrators, clinical pharmacists, nurses, as well as support staff [22]. Fifty-one staff interviews and ethnographic observations from these sites were analyzed to identify and understand the relationships among contextual elements. Research questions were based on the PARIHS model and included questions on staff perceptions of the evidence as well as contextual questions about leadership behavior, leadership culture, local opinion leaders, teamwork, local resources and communication. All interviews were recorded and transcribed. Table 4 describes study participants.

**Table 4:** **Sample characteristics**

**Table of sites and participants 2012**	
**Feature**	**N**
Number of sites	5
Number of staff interviewed	51
Chief	4
Middle managers (Associate chief, clinical coordinator, etc.)	4
Pharmacist	38
Pharmacy technician	1
Nurse	1
Clerk/Health technician	3
Average number of participants per site	7.4
Average length of interview	39 minutes

Two trained qualitative observers shadowed clinic staff as they engaged in delivery of care including but not limited to, work processes, job tasks and roles, and social dynamics at each ACCII site for at least an entire day and often two days. Observations were collected in the form of field notes. Field notes are written records made during site visits or immediately thereafter. The Institutional Review Board (IRB) of the Bedford VA Medical Center approved the study.

### Data analysis

Qualitative interview transcripts were formatted and imported into NVivo 10 [QSR International, Australia] for data management, coding and analysis. An *a priori* coding scheme guided by PARIHS to identify information on evidence, context and facilitation was developed. We also used an iterative thematic analysis so that we could capture any interesting relationships, patterns, surprises, and inconsistencies among people and within and across sites [23]. Two researchers separately performed open coding of seven transcripts which led to refinement of the *a priori* codes from PARIHS and identification of emergent content and themes for additional codes. This coding resulted in the development of a codebook with definitions and exclusion and inclusion criteria. Three members of the research team completed coding of the interviews. A subset of interviews was independently double-coded to affirm inter-coder reliability [24]. Observational field notes were read independently by the research team. The qualitative team then applied the code book from the interviews to the field notes and linked the field notes to interview coding through the use of the memo feature in NVivo 10.

Members of the qualitative team met regularly to review coded texts and resolve discrepancies through consensus as well as identify and resolve within site and cross-site variation in themes. The qualitative team also used the same process to resolve any discrepancies in our definitions and characterizations contextual elements at each site and our characterizations of the strength of contextual elements in regard to the ACCII (Table 1) and our description of uptake strength (Table 5).

**Table 5:** **Definitions of uptake levels**

**Uptake levels**	**High**	**Medium**	**Low**
**Uptake component**			
ACC dosing algorithm	*Algorithm is implemented and used a high percentage of the time by all staff	*Algorithm is inconsistently used among staff	*Algorithm is rarely used among all staff
Dashboard	*Dashboard is used not only to measure performance but as tool for targeting poor TTR patients for more monitoring	*Dashboard used to measure performance inconsistently and only one or two features used inconsistently as a tool	*Dashboard used rarely to measure performance and rarely or not at all as a tool
Site specific QI work	*Initiated site specific improvements	*Site has thought of improvements but inconsistent initiation and follow through	*Site demonstrates no initiative and attempts few or no improvements
	* Shares results	*Staff inconsistently participate	*Staff rarely or never participate
	*Staff regularly participate		
Site seeks out and/or accepts facilitation by ACC improvement team	*Site reaches out for assistance and responds to ACC improvement team	*Participation is inconsistent	*Site does not reach out or respond
Participation in local ACC coordinators leadership team run by ACCII	*Site participates-attends meetings	*Site mostly participates-attends most but not all meetings	*Site often does not participate-attends meetings unevenly
	*Leader facilitates ACC coordinator participation	*Leader facilitates ACC coordinator participation most of the time	*Leader does not always facilitate ACC coordinator participation
TTR (Time in therapeutic range)	*TTR begins to improve	*TTR shows some movement but not much	*TTR shows no improvement

## Results

The degree of evidence-based practice (EBP) uptake varied across sites and we found interaction among contextual elements was a major factor influencing uptake. In this section, we provide site summaries which describe the state of contextual elements in site-specific case study tables (Tables 6, 7, 8, 9 and 10). This site has a single, unified ACC workforce, which manages patients for a medical center and two large affiliated community-based outpatient clinics (CBOCs) (Table 6). Site B had a central medical center and many CBOCs. This ACC has a goal of centralizing the ACC to share patients, workload and labor (Table 7). Site C was centralized and used a unified workforce to manage patients from a medical center and affiliated CBOCs (Table 8). Site D was part of a large medical center whose ACCs are not unified into a single clinic; there is no pooling of labor or workload (Table 9). Site E was also part of a large medical center that while not centralized entirely has campuses that communicate and do occasionally share workload (Table 10). Then we present our analysis of how these contextual factors interact with each other. Attitudes toward evidence are included in each site summary because this paper explores how contextual factor interplay influences uptake of EBPs. An asterisk has been placed by the contextual element that was considered the strongest.

**Table 6:** **Site A summary**

**Evidence**	Teamwork*	**Communication**	**Leadership**
-Already had a practice algorithm	-Culture of improvement where staff made suggestions	-In constant communication about workload	-New leader
-Knowledgeable and comfortable with EBPs and working with algorithm.	-Organized and cooperated with each other	-Communicated about patients and patient issues	-Soon became an active supporter of the ACCII
	-Knew each other’s strengths and weaknesses	-Used all means available to talk (email, phone, Lync messenger, face-to-face)	-Always lets staff attend ACCII meetings
	-Worked together for common good	-Site designed tracking system to manage patients before ACCII started	
	-Team cited teamwork as their strength		

*Most influential contextual factor.

**Table 7:** **Site B summary**

**Evidence**	**Teamwork**	**Communication**	Leadership*
-Already had a practice algorithm but did not use it or refer to it consistently	-Teamwork generally perceived as adequate, but staff somewhat divided	-Functional but uneven communication about patients and workload	-Recent promotion of ACC pharmacist to middle manager (MM)
-Staff was unevenly open to a new algorithm	-Less cohesiveness as a team	-Used all means available to communicated (email, phone, IM, face to face)	-MM very supportive of ACCII
-Concern about losing clinical judgment if just following an algorithm	-Less willingness to pitch in to even out the work load	-Deeper level of communication about quality improvement often lacking at outset	-MM interested in QI approaches & solicited staff ideas
	-Uneven interest in change and improvement	-Site designed tracking system to manage patients before ACCII started	-MM supported local coordinator (liaison to ACCII)
			-MM supported by pharmacy leadership

*Most influential contextual factor.

**Table 8:** **Site C summary**

**Evidence**	**Teamwork**	Communication*	**Leadership**
-Strain of skepticism about the algorithm	-Organized, cooperated with each other	-Communicated about workload and patients	-Middle manager (MM) supportive of ACC team
-Belief in clinical judgment of clinical pharmacists	-Knew strengths and weaknesses	-Staff noted communication as a strength	-MM vocalizes skepticism of algorithm; staff are aware
-Algorithm thought to be good only for training inexperienced staff	-Worked together for common good	-Used all means available to communicate (email, phone, IM, face to face)	-Staff have respect for MM
	-Interested as a group in change and improvement	-Team was safe space to express ideas and concerns	-MM supports sending a staff person to ACCII meetings
	-Team itself cites teamwork as their great strength	-Communication between ACC staff and leadership effective	-MM had support of pharmacy leadership

*Most influential contextual factor.

**Table 9:** **Site D summary**

**Evidence**	Teamwork*	**Communication**	**Leadership**
-Negative attitude toward algorithm	-Organized &cooperated with each other	-Adequate communication about workload and patients	-Middle manager (MM) did not micromanage and allowed fair amount of autonomy to ACCs
-Great belief in clinical judgment of clinical pharmacists	-Worked together for long time	-Used all means available to talk (email, phone, IM, face to face)	-MM noted there is change fatigue
-Discomfort with being asked to adopt an EBP	-Strong identification as a team with shared values and practices.	-Communication between ACC team and leadership was rare	-MM remained noncommittal regarding support of the ACCII
	-Relatively unaware of ACCII project		
	-Team itself cited teamwork as their great strength		

*Most influential contextual factor.

**Table 10:** **Site E summary**

Evidence*	**Teamwork**	**Communication**	**Leadership**
-Had not used an algorithm to date	-Not very organized and low cooperation overall	-Low level of intra-ACC staff communication	-Staff feel unevenly supported by leadership
-Very interested in getting some guidance	-Internal divisions where some team members cooperate, but not others	-Used all means available to talk (email, phone, IM, face to face)	-Staff felt poorly informed about ACCII
-Receptive to algorithm	-ACC staff note that they do not have great teamwork	-ACC staff note that communication among ACC team is strained	-Sense from staff that leadership is not very interested in ACCII
-Concerned about workload implications of the 7 day return for out of range patients recommended by the algorithm		-Communication between ACC team and leadership was rare—ACC team had heard very little about the ACCII	-Leadership supported sending staff to ACCII meetings

*Most influential contextual factor.

### Cross-site interplay findings

Our findings demonstrate how interaction among contextual elements, like teamwork, communication and leadership, led to what seemed to us to be relatively consistent patterns with regard to the impact on uptake. In the context of our implementation effort, we ranked the contextual elements as strong, medium and weak (Table 1) in terms of whether or not they were conducive to our intervention (ACCII). We carefully characterized what constituted successful, medium and low uptake of the ACCII in Table 5. Table 2 summarizes how interactions among contextual elements produced different effects on levels of uptake, some of which were unexpected. In Table 3, we ranked the impact of each element on the ACCII (negative, moderate/neutral, and positive) and concluded by listing overall impact the interplay among elements had on uptake of the ACCII.

We observed three general patterns of interaction among contextual elements and evidence. First, belief in the evidence, while necessary for uptake, was not sufficient. Second, we observed that while belief in evidence was important, a site needed at least one other contextual element to be strongly favorable, be it leadership, teamwork, or communication, for uptake to occur. In contrast, if a site had unfavorable context in all three of areas, successful uptake was unlikely to occur. Third, we observed that when belief in the evidence was limited, even noteworthy strengths in other areas only served to reinforce the determination to resist any change to embrace the EBP. Details about these interaction patterns are found below.

One of our most striking findings was that belief in and comfort with the evidence, while important, was not by itself a determining factor in ACCII uptake. Sites A (Table 6) and B (Table 7) perceived the evidence to be quite strong, but at Site A, teamwork and communication facilitated the uptake, while at Site B, leadership supported uptake. In neither case was belief in the evidence alone sufficient to initiate and maintain a high level of ACCII uptake. At Site A, strong teamwork and communication were combined with a strong belief in the evidence, which led to a high rate of uptake. In part this was because site A has strong teamwork and communication skills. One pharmacist remarked,

*“We have very strong communication…We try to be very thorough in our notes so that the next person picking it up will know exactly where it was left off, what the story is…. We use (Microsoft) Communicator. We use the phone and we use the spreadsheet to share info…and I think that we’re really good about talking to each other about difficult cases and that kind of helps us to make the best clinical decisions that we can. …we work really well as a team…we talk a lot and we communicate about what’s going on”.*

Site A already had a culture of improvement, as seen in the following quote:

*“We are looking to optimize the process that we use… I think it’s a fabulous idea. I’m looking for ways that we can improve… so you are less likely to make mistakes or worry about making mistakes … what would be wonderful is if we could figure out who’s doing a better job with that kind of thing and how can we implement it here”.*

Having a new leader who needed time to adjust and learn on the job was not a barrier to change for Site A, because of their strong teamwork and communication.

Site B had generally adequate teamwork and communication, which was not without its weaknesses however. One staff member remarked of team work and communication at Site B,

*“…It’s really individual specific…For some folks it’s…really good. Others, there’s a chronic lack of communication and lack of…intuition or empathy, you know, as far as what other people’s schedules are and… [this] impacts other people’s function”.*

It was through the enthusiastic leadership of a new middle manager that Site B had high levels of uptake in the ACCII. One staff person remarked,

*“[The middle manager] is so organized… [The manger] is wonderful”.*

Another stated:

*“[MM] is just so helpful and…she really understands the issues that we face and, I mean, she is obviously on board with anticoag project…”*

Teamwork and communication was mediocre at Site B, but in this case it was strong leadership, and a team willing to be led, that changed the contextual interplay dynamics and enabled the site to become a site with high uptake. In this case, the interaction among contextual elements that varied in strength nevertheless led to high uptake. Here again, we see that at least a moderate belief in the evidence, combined with at least one strong contextual factor (for Site A, teamwork and communication, and for Site B, leadership) were sufficient to enable successful implementation to occur.

At Site C (Table 8), many elements of context seemed relatively favorable, as the site had relatively strong teamwork and communication, in addition to a highly able leader. However, a closer examination revealed that this leader, while highly able and beloved, harbors a skeptical attitude about the evidence underlying the algorithm. This leader did not actively stand in the way of change, and there was partial uptake at Site C, but its extent was limited in large part due to the skepticism toward the evidence shared by leadership with staff. Although we have rated leadership as “moderate” at this site in Table 2, this does not mean that this leader’s influence was moderate– in fact, the leader was extremely influential and this had a mixed impact on our ACCII (see Table 3). A staff member said of the algorithm,

*“…I’m kind of willing to give it a shot. But …I think we might have some other pharmacists here that might want to use the clinical judgment card pretty frequently on that one”.*

They were referring to their clinical leader. Teamwork and communication were strong at this Site C. One pharmacist stated:

*“…We’re such a good team, we really work together to help everybody and I think when you respect the person that you’re working with and that feeling is mutual I think it makes the clinic run a lot smoother. You know, I think patients are satisfied and… We really do work together as a team”.*

Staff made it clear that they felt their leader was very much a member of the ACC team. A newer staff member notes that intra-team communication was great and that communication with their leader was excellent as well. In fact this staff member noted,

*“… if I have a question…Like, procedural stuff a lot of times I’ll ask…. If I have a quick question about…dosing or something like that, a lot of times I’ll ask [the middle manager]”.*

Staff felt comfort with and respect for their middle manager. However, this site remained at a mid-level of uptake initially in part because of this leader’s lukewarm response to the EBP, resulting in only partial fidelity to the ACCII. Site C illustrates the pattern where if belief in the evidence is lukewarm, even strengths in other areas cannot entirely compensate enough to improve uptake.

At other sites, there was an open distrust of EBPs; we observed that uptake was limited in proportion to the extent of the distrust. At Site D (Table 9), distrust of the evidence for our EBP was high. Furthermore, the leadership at Site D also did not seem interested in promoting the ACCII. An interviewee indicated that participation in the ACCII was entirely optional despite the fact that the algorithm was mandated.

A participant at Site D referred to anticoagulation care as an “art” more than a “science,” indicating a discomfort with the EBP. This pharmacist explains,

*“…to me you can’t categorize every patient into that algorithm. They don’t all fit and a lot of times it’s a gut feeling that you need to change a dose”. *

In the context of such dismissal of evidence, the strong teamwork and communication present at Site D only served to reinforce resistance to the effort to implement ACCII. In regard to teamwork, one team member stated,

*“… [We] know that we can depend on each other and anyone’ll back you up and help you out”.* Another noted

*“We all [have]… a similar philosophy, a similar training and a similar mindset, so I think our team play [emphasis in interview] works well…”*

And another staff member noted that they anticipated that their site would be, *“the most resistant to change”.* In discussing why there was resistance to change, there was a celebration of this team’s closeness. For example, in the past, there had been an effort to standardize one aspect of ACC practice at this site. This local QI effort met with no success at Site D as seen in almost identical quotes by two Site D team members,

*“Well we didn’t want to use that [the proposed QI tool]…and I don’t remember actually my own feelings about it but I remember that you know as a team we kind of shut it down…we decided to just stay with our way….”*

Another staff member said,

*“We didn’t want to use it [the proposed QI tool]…as a team we… said no”.*

Communication and teamwork worked together to prevent uptake of a local QI project. Leadership engagement and communication about the fact that the ACCII was being implemented had not even reached all staff when the research team began interviewing. A staff person said,

*“I don’t keep my ear that close to the ground so, I just sit back and wait for official things to come…Just [heard from leadership] that it’s… [ACCII] coming…It’s [ACCI] in the works…”*

Since leadership had a “hands-off” approach to the running of the ACC at Site D, staff felt that participation in the ACCII was not required and therefore they either did not participate or participated reluctantly and infrequently. At Site D (Table 9), the research team ranked leadership as “weak” because leadership did not support the implementation of the ACCII but Site D staff felt that their leader was strong and influential (Table 2). Therefore, leadership at Site D had a negative impact on the ACCII and this is reflected in Table 3

At Site E (Table 10), we found solid belief in the evidence but the relative absence of any other strong contextual factors resulted in very limited uptake at this site. Site E is a good counter-example to high uptake sites such as A and B. At Sites A and B there was solid belief in the evidence but there was also strong contextual factors that facilitated uptake. At Site E this was not the case. Most staff members at Site E did express a clear belief in the evidence underlying the algorithm that our project promoted. For example, a pharmacist observed,

*“…I have seen a change in people’s attitudes towards…using the algorithm. I like it. [It] takes a little bit more time when we’re using [the algorithm] because we’re kind of dosing a little bit differently than we had in the past…and you kinda have to think things through a little bit more which isn’t a bad thing”.*

However, other contextual factors were notably weak. Teamwork at Site E is described by a staff member as,

*“It [teamwork] is awful …There are certain people that will not be in a team, no matter how hard you try…I would love to have a team atmosphere and be able to count on everybody and feel like there’s trust and that we’re providing the best care, but we do not have that here”.*

The feeling was that poor intra-team relations and communication impacts work performance. Furthermore communication between leadership and the ACC staff also impacted staff attitude toward their teamwork, their work and their attitude toward change. A Site E pharmacist reported,

*“…the other thing is communication…we don’t have staff meetings. We…just get emails…but nothing is really discussed among the group”.*

This was reinforced by another staff member*;*

*“I think the hardest part of our job is communication…. the volume of our workload is very high so that’s challenging. We have so many patients that walk right in our door. …I think another challenge is the lack of communication among the pharmacists. With the clinical staff [some] have meetings with our [leadership] …they knew about all these…changes, however it doesn’t get trickled down to the rest of the clinic… we’re not [the entire staff] informed as to what’s happening”.*

As to leadership, the picture is generally one of frustration. Some staff note that that the middle manager is, *“…looking at what can we do better. But anyone above him, we don’t see, we don’t hear from”.* The following quote illustrated general staff attitude in this area:

*“…People here want to always make an impression, okay? Especially the people in management… They come around when there’s the photo op, I always like to say… On a daily basis, forget it, okay? That’s the way it is, you know”.*

Here, we see that even a site with a positive view of the evidence cannot succeed when context was very weak.

## Discussion

Theoretical frameworks in implementation science and related fields generally state that several contextual elements contribute to successful implementation, defined as full and high-fidelity uptake of an EBP. While interactions among these contextual elements have been tacitly assumed to occur, they have rarely been described in detail. Our study examined five sites as examples of how contextual elements may interact with each other and with perceptions of the evidence to promote or inhibit adoption of an evidence-based practice. We observed that no single shortcoming served as a “fatal flaw,” but that three general interaction patterns emerged from our data that mapped with successful or unsuccessful uptake of the ACCII.

1. Evidence alone was not sole determining factor in uptake.
2. A site needed at least one and possibly two contextual elements to be strong (such as leadership, teamwork or communication) for uptake to occur. If a site was relatively weak in all three of these, uptake would not occur, despite strong belief in the evidence.
3. If belief in the evidence was limited, strengths in other areas often did not work in favor of uptake, but rather served to reinforce the status quo (i.e. resistance to change).

With the recent emphasis on theory-driven implementation studies, theory has seemed to diverge regarding whether to focus on the key role of individuals in uptake of interventions, or whether to shift attention to the roles of teamwork and social networks [12]. In part this may be because the relationships among team members appear to be of key importance; often, but not always, surpassing the importance of individual attributes [25]. What interactional data taught us, however, was that both suppositions have merit. Relationships among team members were a very important contextual dynamic that impacts uptake, but individual actors were also of key relevance.

If there is a contextual element that is particularly strong, this strength may compensate for weaker areas, as was seen with Site A (Table 6). Site A had a strong belief in the evidence and was strong in teamwork and communication but moderate in leadership simply because the leader was new. Nevertheless high uptake resulted. Conversely, when all contextual elements were poor, uptake was predictably low even if there was belief in the evidence. Site E (Table 10) was an example of this. Many sites had more of a mixed picture, with contextual factors being strong in some areas and weak or moderate in others. If the evidence was accepted, even moderately, and one other contextual factor was strong or “good enough”, uptake happened. Site B (Table 7) was an example of this type of interplay. Another operative pattern was that if evidence was considered weak, even what would ordinarily constitute strengths in other contexts does not work to help facilitate uptake. Sites C (Table 8) and D (Table 9) respectively are examples of partial and low uptake. In some cases, even potential strengths (such as a high degree of teamwork) were turned into weaknesses (e.g. a tight-knit team working together in close cooperation against EBP), as occurred at Site D.

Many conceptual frameworks present taxonomies of contextual elements which can be used prospectively to guide a theory-driven implementation, to tailor interventions to address perceived needs on an ongoing basis, and to analyze implementation retrospectively. In our study, PARIHS was used prospectively as part of the overall study design which mapped out how the research team would study the implementation as it rolled out, developed and spread. Specifically, the research team drew on PARIHS to construct the interview guide and to develop the code book for the interviews and ethnographic observations. However, in the course of collecting and analyzing our data we realized that we were encountering a limitation in our conceptual model. PARIHS did not provide us a way to describe and the interplay among contextual elements, nor did it represent how contextual interrelationships influenced initial rates of uptake. In this study, we learned that interactions among contextual elements can produce a synergy which magnifies contextual effects and moves them in certain patterns, some of which we were able to characterize here.

We operationalized the patterns we observed in the qualitative data by feeding the information back to the ACCII facilitation team and working with them to tailor the intervention to be site specific. Some of this tailoring took the form of doing some targeted capacity building and development of contextual elements at sites to enhance uptake. For example, there were acts of facilitation such as teamwork development, encouraging and facilitating better communication within and across sites about the ACCII, and reaching out to leadership to enlist support for the intervention that came directly out of the research team’s rapid site assessment. Additionally there were increased efforts in algorithm presentations, journal clubs and other venues to educate ACC staff about the scientific basis of the algorithm and the importance of its use, especially where belief in the evidence seemed to be the weakest link.

As a concrete example of the value added by our approach to analyzing not only contextual elements, but also their interaction, the research team observed that teamwork was likely to be the “weak link” at Sites D and E. The team was “weak” at Site D – with regard to our intervention – because they worked so well together and mutually reinforced each other’s views of the limited value placed ACCII. In this case, team cohesion created resistance that negatively impacted implementation of the intervention. The team at site E was weak because they did not know how to work toward a common goal, they did not support one another and they did not have established means of communicating about common goals because they did not hold regular meetings. Our assessment of teamwork as the key factor driving successful implementation at these sites was in part predicated on our understanding that “unlocking” teamwork would also enable other aspects of context to improve. Informed by this observation, both the research team and the facilitation-implementation team concentrated on team-building activities at Site E, and on trying to convince team members at Site D regarding the evidence for the algorithm, as well as reaching out to the Site D leadership to educate them. Based on our interim findings, our efforts seem to be making a difference at both Sites D and E, and indeed focusing on teamwork first at both sites (albeit in different ways) did enable other aspects of context to improve subsequently. This serves as a concrete example of how our interactional approach to data analysis also helped us tailor the intervention more effectively.

Quality improvement (QI) does not rest on the intervention itself but often on the context in which the intervention takes place. For example, one recent study examined how contextual elements interacted in the setting of a single-site intervention to reduce surgery cancellations among 20 staff [7]. In that study, the authors drew on the Model for Understanding Success in Quality (MUSIQ) framework to identify three common themes about how contextual elements influenced change. These three contextual themes were: (1) identifying the need to change, (2) facilitating system-wide improvement, and (3) leader involvement and support [7]. Our study greatly extends findings of this study, and provides five different examples of how contextual elements can interact to promote or inhibit uptake of an EBP. Further studies of the interaction among contextual elements of uptake will also be essential, as we begin to build a theoretical framework not only of the determinants of successful implementation, but also how contextual and domain interactions enhance or inhibit implementation. We expect that future studies will confirm some of our findings, but also will perhaps uncover some other important patterns of interaction among contextual elements, in addition to the three patterns that we describe here.

Our study’s cross-site analysis suggests that the relationship between evidence, contextual factors and uptake is a very mixed picture but that there are common patterns among interactional data that influence uptake (Tables 2 and 3). Future studies may contribute additional case studies, which can broaden our understanding of how such elements interact; with additional observations, certain patterns may emerge and repeat themselves. The reproducibility of our findings in future studies will thus be of great interest.

### Limitations

This study has some limitations. Most importantly, interactional data can be challenging to identify and discuss as they are based on social actions and actors who are always changing. We added ethnographic observations, which allowed for a more detailed understanding of interactional data, to ameliorate this limitation.

## Conclusion

Interrelationships among different contextual elements can act as barriers to uptake at some sites and as facilitators at others. Accounting for interconnections among elements enables PARIHS to more fully describe the determinants of successful implementation as they operate in real-world settings. Our study is an important start to better understand these interactions, which have been proposed and assumed, but rarely described in detail. This approach to understanding the interaction of contextual elements also has the potential to help inform a more effective approach to tailoring the intervention to promote successful implementation.

## Abbreviations

ACCII: Anticoagulation Clinic Improvement Initiative
ACC: Anticoagulation Care Clinic
EBP: Evidence-based Practice
IRB: Institutional Review Board
IM: Instant messaging
IS: Implementation Science
MM: Middle manager
MUSIQ: Model for Understanding Success in Quality
PARIHS: Promoting Action on Research Implementation in Health Services
QI: Quality Improvement
VA: Department of Veterans Affairs
VISN: Veterans Integrated Service Network
